# Supplementary material for: MOTUM: A system for Motion Online Tracking Under MRI
Source: Imaging Neurosci (Camb). 2026 Jan 7;4:IMAG.a.1081. doi: 10.1162/IMAG.a.1081 (PMC12779753; doi:10.1162/IMAG.a.1081)
Supplement: Supplementary Figure 1 [file IMAG.a.1081_Figure_1.pdf]

Supplementary Figure 1. Magnet room spatial configuration and control system wiring diagram

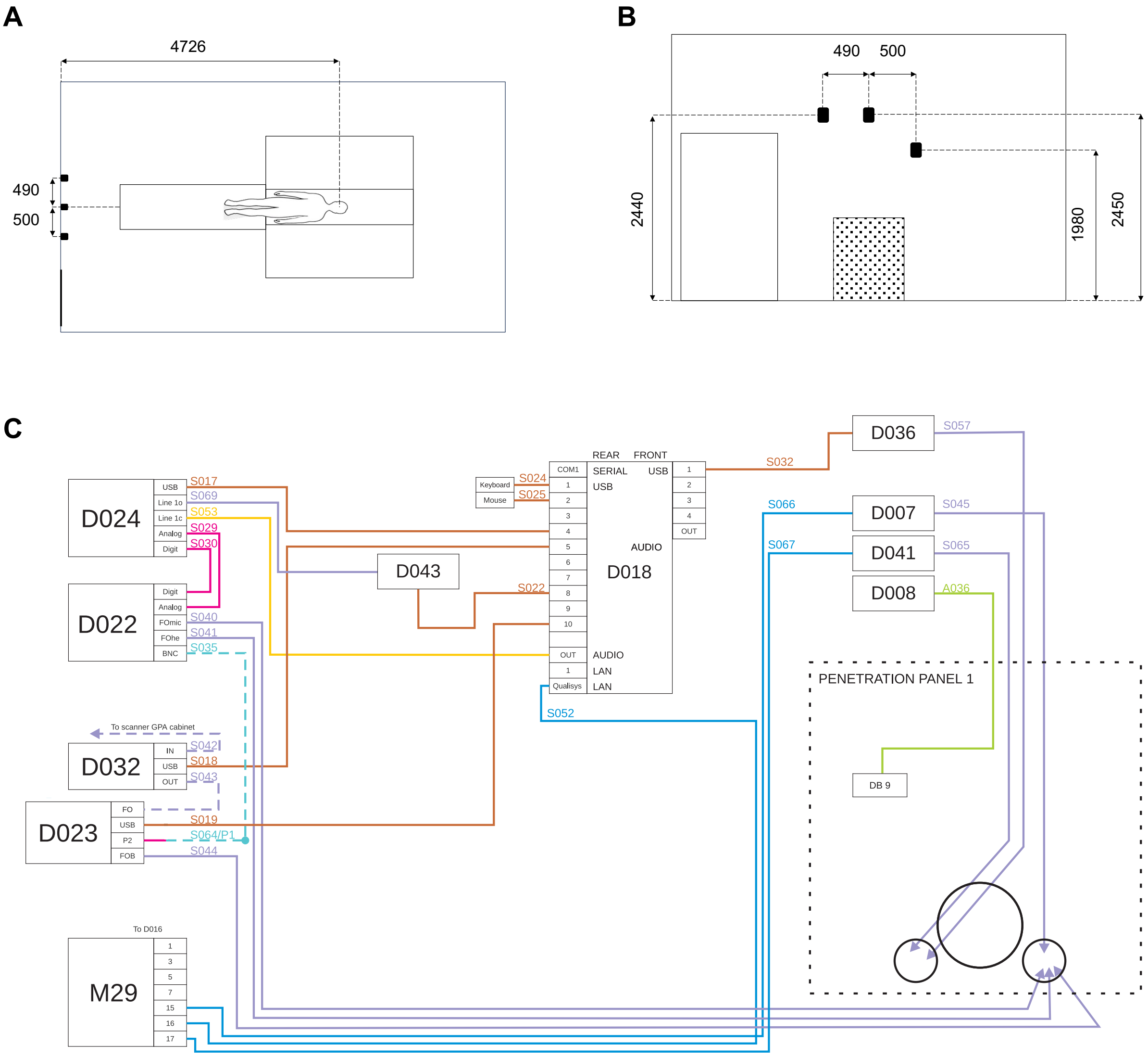

(A) Top view and **(B)** frontal view of the magnet room showing the positions of three Qualisys motion capture cameras. The central camera was mounted at 2450 mm height, centered horizontally with the magnet isocenter; the left camera at 2440 mm height and 490 mm lateral offset; and the right camera at 1980 mm height and 500 mm lateral offset. Dimensions are shown in millimeters. **(C)** Schematic representation of devices and connections in the control room. Main line power supply and screen connections are omitted; only devices relevant to the experimental setup are shown. Lines: coaxial (cyan), optical fiber/FO (purple), custom copper connection (magenta), LAN (blue), USB (brown), coaxial audio (yellow); trigger lines are dashed. Devices: D007, Qualisys FO/copper converter #1; D008, Qualisys cameras power supply; D018, control PC; D022, Optoactive Digital Signal Processor; D023, Current Designs 932 trigger and response devices interface; D024, Optoactive console; D032, Siemens trigger converter; D036, gloves interface; D041, Qualisys FO/copper converter #2; D043, FO audio interface; M29, LAN switch. Ports are represented by small rectangles on device borders and are labeled/numbered accordingly. The magnet room penetration panel in the control room is shown as a black dashed square; circles in the lower part represent wave guides, and the DB9 connector is lowpass-filtered. Trigger signal flow: the FO trigger signal is converted by D032 to a fixed-width pulse (10 ms) and delivered to D023, which distributes the trigger pulse via USB (through keyboard emulation) and coaxial lines; USB trigger is received by D018, while coaxial trigger signals are received by D022 (for sequence synchronization). Audio signal flow: audio generated by D018 is delivered to D024/D022 via both copper and FO media (the latter through D043 interface).
