# Supplementary material for: MOTUM: A system for Motion Online Tracking Under MRI
Source: Imaging Neurosci (Camb). 2026 Jan 7;4:IMAG.a.1081. doi: 10.1162/IMAG.a.1081 (PMC12779753; doi:10.1162/IMAG.a.1081)
Supplement: Supplementary Figure 2 [file IMAG.a.1081_Figure_2.pdf]

Supplementary Figure 2. Cue images of the four hand poses presented to participants for kinematic glove calibration

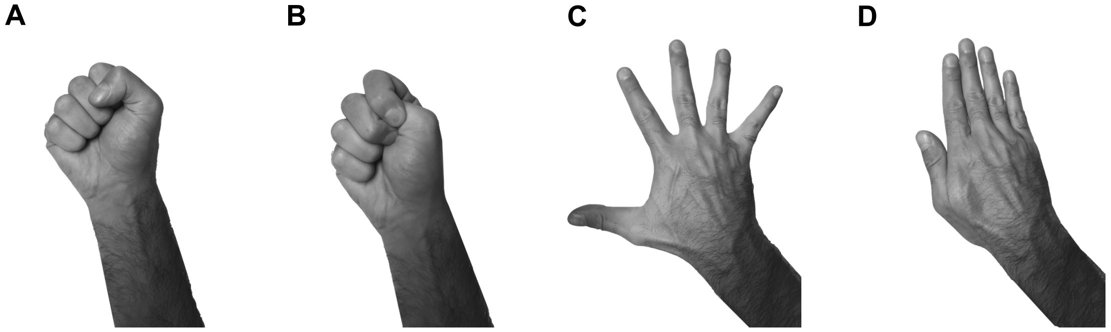

(A) Closed fist with the thumb bent over the index and middle fingers. **(B)** Closed fist with the thumb folded into the palm and covered by the fingers. **(C)** Open hand with fingers fully extended and spread apart. **(D)** Flat hand with fingers extended and held close together.
