# Supplementary material for: MOTUM: A system for Motion Online Tracking Under MRI
Source: Imaging Neurosci (Camb). 2026 Jan 7;4:IMAG.a.1081. doi: 10.1162/IMAG.a.1081 (PMC12779753; doi:10.1162/IMAG.a.1081)
Supplement: Supplementary Figure 3 [file IMAG.a.1081_Figure_3.pdf]

Supplementary Figure 3. Framewise displacement (FD) timeseries across all scanning runs

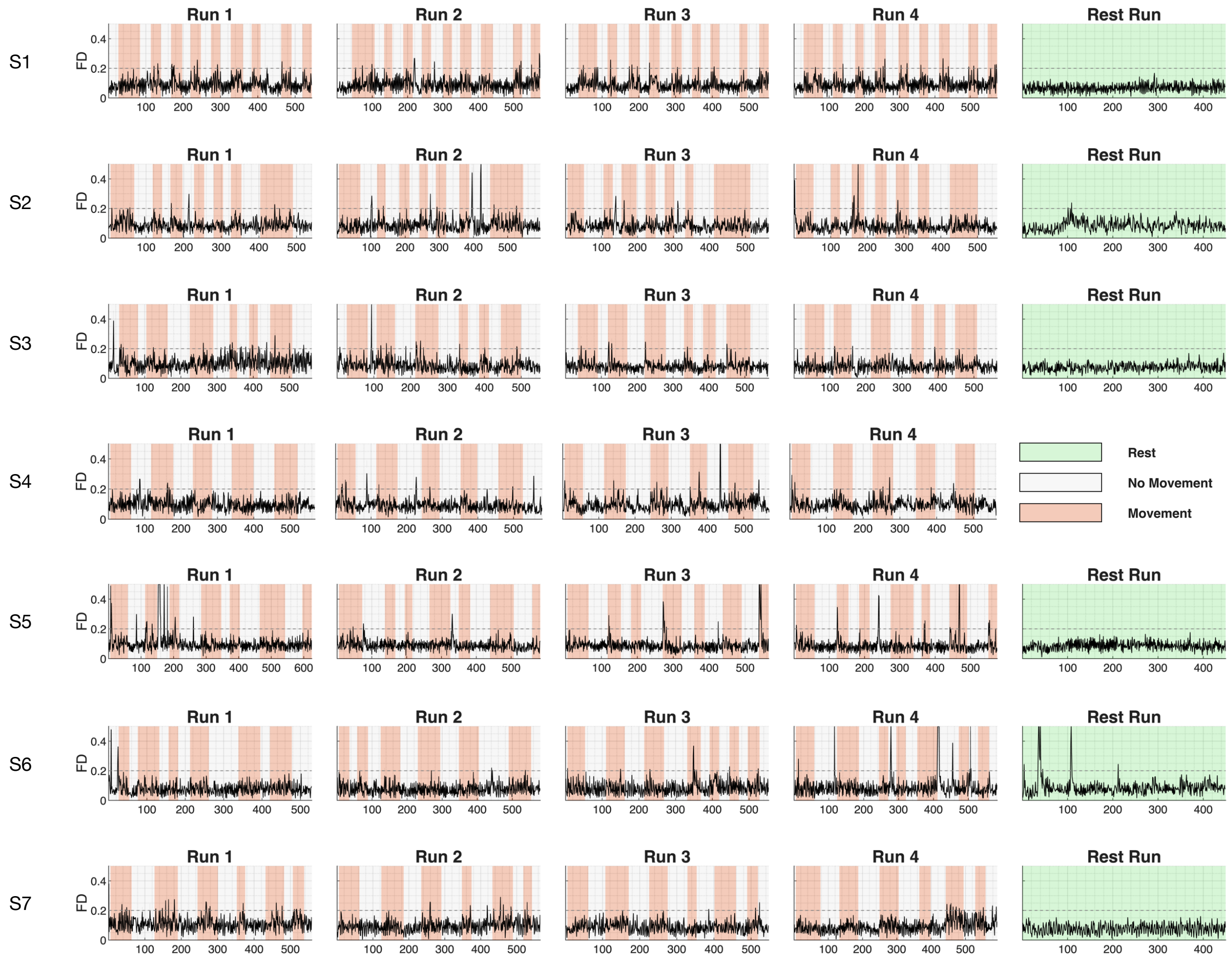

Each row represents a different participant, with volume-wise FD values plotted on the y-axis (maximum 0.5 mm). Background colors indicate scan conditions: orange for “Movement” blocks, white for “No Movement” blocks, and green for resting-state scans. The dashed horizontal line at 0.2 mm represents the optimal FD threshold reported in the literature, while 0.5 mm indicates the acceptable upper limit according to Siegel et al. (2014). Note that S5 did not complete the resting-state scan.
