# Supplementary material for: MOTUM: A system for Motion Online Tracking Under MRI
Source: Imaging Neurosci (Camb). 2026 Jan 7;4:IMAG.a.1081. doi: 10.1162/IMAG.a.1081 (PMC12779753; doi:10.1162/IMAG.a.1081)
Supplement: Supplementary Table 1 [file IMAG.a.1081_Table_1.pdf]

Supplementary Table 1. List of the kinematic measures and results of the principal component analysis showing their loadings onto the seven extracted principal components described in Table 1.

Phase	Kinematic measures		Principal components						
			1	2	3	4	5	6	7
Reaching phase	Duration			0.706					0.327
	Arm	Distance traveled	0.702	0.558					
		Average velocity	0.638			0.576			
		Maximum velocity	0.652			0.569			
		Curvature index							0.852
		Maximum deviation	0.433	0.699					
	Hand	Distance traveled		0.856		0.329			
		Average velocity				0.915			
		Maximum velocity		0.353		0.838			
		Curvature index							0.835
		Maximum deviation		0.814					
Back-movement phase	Duration				0.369		-0.611		
	Arm	Distance traveled	0.821		0.388				
		Average velocity	0.608				0.620		
		Maximum velocity	0.721				0.499		
		Curvature index							0.689
		Maximum deviation	0.618		0.483				
	Hand	Distance traveled			0.915				
		Average velocity					0.818		
		Maximum velocity			0.575		0.667		
		Curvature index							
		Maximum deviation			0.826				
Grip phase	Grip aperture	Average aperture						0.899	
		Minimum aperture						0.825	
		Maximum aperture		-0.360				0.697	
	Hand aperture	Average aperture					-0.440	0.707	
		Minimum aperture					-0.531	0.649	
		Maximum aperture		-0.505				0.510	
