# Supplementary material for: MOTUM: A system for Motion Online Tracking Under MRI
Source: Imaging Neurosci (Camb). 2026 Jan 7;4:IMAG.a.1081. doi: 10.1162/IMAG.a.1081 (PMC12779753; doi:10.1162/IMAG.a.1081)
Supplement: Supplementary Table 2 [file IMAG.a.1081_Table_2.pdf]

Supplementary Table 2. Explained variance in regional brain activity by arm and hand framewise displacement

Region	Arm framewise displacement			Hand framewise displacement		
	Mean %	Min %	Max %	Mean %	Min %	Max %
M1	0.70	0.05	1.86	1.21	0.08	4.88
PMd	0.61	0.00	2.67	1.12	0.00	5.28
PMv	0.42	0.00	1.22	0.70	0.01	1.83
SMA	0.64	0.00	2.74	0.95	0.02	5.11
SPL	0.44	0.00	1.19	0.46	0.04	1.47
IPS	0.37	0.05	1.29	0.54	0.04	1.54

Percentage of residual variance (R^2) correlating with arm and hand framewise displacement regressors. Values show mean, minimum, and maximum across seven participants for left-hemisphere motor ROIs (M1: primary motor cortex, PMd: dorsal premotor cortex, PMv: ventral premotor cortex, SMA: supplementary motor area, SPL: superior parietal lobule, IPS: intraparietal sulcus).
