# Supplementary material for: MOTUM: A system for Motion Online Tracking Under MRI
Source: Imaging Neurosci (Camb). 2026 Jan 7;4:IMAG.a.1081. doi: 10.1162/IMAG.a.1081 (PMC12779753; doi:10.1162/IMAG.a.1081)
Supplement: Supplementary Table 3 [file IMAG.a.1081_Table_3.pdf]

Supplementary Table 3. Average effect of single PCA kinematic components on regional brain activity

Condition		Principal component	M1	PMd	PMv	SMA	SPL	IPS
Move Invisible	1	Arm velocity	0.14	0.27	0.33	0.18	0.32	0.37
	2	Distance (reaching)	-0.05	0.00	-0.28	-0.06	-0.09	0.09
	3	Distance (back movement)	-0.10	-0.04	-0.18	-0.08	0.07	0.11
	4	Arm and hand velocity (reaching)	-0.11	-0.26	-0.17	0.09	-0.24	0.01
	5	Arm and hand velocity (back movement)	0.11	0.19	0.18	0.50	0.06	0.13
	6	Grip and hand aperture	-0.40	-0.10	0.38	-0.30	-0.17	0.38
	7	Trajectory curvature	0.72	0.14	-2.75	-0.30	-0.32	0.62
Move Visible	1	Arm velocity	-0.07	-0.06	0.11	-0.44	-0.16	0.22
	2	Distance (reaching)	0.26	0.28	0.31	0.39	0.17	0.02
	3	Distance (back movement)	-0.29	-0.11	-0.22	-0.21	0.01	0.37
	4	Arm and hand velocity (reaching)	-0.15	-0.05	0.05	0.22	-0.29	0.01
	5	Arm and hand velocity (back movement)	-0.35	-0.16	0.06	-0.04	0.31	0.03
	6	Grip and hand aperture	-0.61	-0.01	-0.43	-0.69	-0.06	0.47
	7	Trajectory curvature	-2.74	-2.20	4.42	2.02	1.98	0.62

Mean regression coefficients (betas) across participants showing how each principal component derived from kinematic measures (see Table 1) modulates BOLD activity during invisible and visible movement conditions in left-hemisphere motor ROIs (M1: primary motor cortex, PMd: dorsal premotor cortex, PMv: ventral premotor cortex, SMA: supplementary motor area, SPL: superior parietal lobule, IPS: intraparietal sulcus).
